# Protective Effects of Zein/Ferulic Acid (FA)–Pectin (PEC)/Chitosan (CS) Nanocomplexes on DSS-Induced Ulcerative Colitis in Mice

**DOI:** 10.3390/foods14132345

**Published:** 2025-07-01

**Authors:** Yifei Guo, Xinyu Yu, Rongrong He, Jianfei Pei, Haiming Chen, Weijun Chen

**Affiliations:** 1 Hainan University-HSF/LWL Collaborative Innovation Laboratory, College of Food Sciences & Engineering, Hainan University, 58 People Road, Haikou 570228, China; gyf1750926027@163.com (Y.G.); 13009436050@163.com (X.Y.); rongronghe@hainanu.edu.cn (R.H.); peijianfei@hainanu.edu.cn (J.P.); 2 Haikou Key Laboratory of Special Foods, Haikou 570228, China

**Keywords:** ferulic acid, nanocomplex, encapsulation, ulcerative colitis, DSS, gut microbiota

## Abstract

Ferulic acid (FA) exhibits beneficial properties in ulcerative colitis (UC) pathogenesis, while sensitivity to the environment and enzymes limits its use in UC therapy. Therefore, this study aims to develop a colon-targeted nanocomplex delivery system using FA and investigate its protective effects and underlying regulatory mechanisms in UC mice. A novel Zein/FA–pectin (PEC)/chitosan (CS) nanocomplex was successfully fabricated in this study. Through systematic adjustment of the PEC/CS-to-Zein/FA ratio, optimal encapsulation efficiency (60.1%) and loading capacity (26.2%) were achieved. The characterized data indicated that hydrogen bonds, electrostatic interactions, and hydrophobic forces were the main driving forces maintaining the formation of the nanocomplexes, accompanied by alterations in the secondary structure of Zein. The Zein/FA–PEC/CS nanocomplexes demonstrated excellent thermal/storage particle size stability and exhibited both protective and sustained-release effects of FA during simulated gastrointestinal digestion. Furthermore, the results demonstrated that the nanocomplexes potentially alleviate UC by regulating inflammatory cytokines, oxidative stress, and gut microbiota. Compared to unencapsulated FA, the nanocomplexes have a better effect on alleviating UC symptoms. In summary, Zein/FA–PEC/CS nanocomplexes have promising prospects in alleviating colitis in UC mice.

## 1. Introduction

Ulcerative colitis (UC) has been classified as a modern refractory disease by the World Health Organization (WHO). Its pathogenesis has been linked to interactions among genetic predisposition, environmental factors, immune response factors, and intestinal microbiota [1]. Conventional therapeutic agents, including 5-aminosalicylic acid (5-ASA), sulfasalazine (SASP), and immunosuppressants, can have side effects and lead to drug resistance [2,3]. Consequently, exploring alternative treatment strategies has become imperative for UC management. Previous studies have revealed that certain food functional factors, such as polyphenols, polysaccharides, flavonoids, and dietary fiber, can prevent and alleviate UC symptoms [4,5,6,7]. Phenolic acids have especially important therapeutic significance in improving inflammatory bowel disease [1].

Ferulic acid (FA) is one of the most abundant phenolic acids in cereals. It possesses multiple bioactivities including antibacterial, anticancer, and anti-inflammatory properties [8]. Numerous studies have demonstrated that FA exerts beneficial effects in alleviating intestinal disorders [9]. FA improved intestinal barrier function by modulating gut microbial composition, thereby alleviating colonic inflammation and tissue damage [10,11]. Evidence revealed that FA can significantly upregulate the expression of tight junction-related proteins, including claudin-1, occludin, and zonula occludens-1 (ZO-1) in human intestinal epithelial cells, which exhibited protective effects on the epithelial barrier [12,13,14]. Furthermore, FA can mitigate colonic inflammation by upregulating regulatory T cells [15]. However, the sensitivity of FA to the environment (heat, light, oxygen, pH) and enzymes limits its efficacy in the treatment of UC. The development of novel FA nanocomposites by constructing a nano-delivery system to enhance the bioactivity of FA and reduce its metabolic consumption during gastrointestinal transport may be an effective strategy to solve the above issues.

In the field of food, protein–polysaccharide complexes have been suggested as the best delivery systems [16]. Common strategies involve modifications using food proteins or polysaccharides to enhance FA stability [17]. Zein, the most abundant storage protein in corn, exhibits amphiphilic and biocompatibility, which has already been utilized to encapsulate or deliver polyphenols [18,19]. Both pectin (PEC) and chitosan (CS) were identified as safe, non-toxic natural polysaccharides with high biocompatibility and favorable biodegradability [20,21]. They remain stable in the dynamic digestive tract and are only degraded by specific polysaccharide enzymes produced by the colonic microbiota [22,23]. This targeted degradation mechanism can reduce premature compound loss in the digestive tract and minimize systemic side effects, which are considered an excellent choice for colonic drug delivery systems. Furthermore, our prior research also confirmed the feasibility of utilizing pectin/chitosan complexes as protective gastrointestinal carriers for the delivery of porous starch-encapsulated bioactive components [24].

In this study, Zein/FA–PEC/CS nanocomplexes were fabricated using the antisolvent precipitation (ASP) method, and the optimal encapsulation efficiency (EE) and loading capacity (LC) were achieved by adjusting the ratio of PEC/CS and Zein/FA. Comprehensive investigations were conducted on the physicochemical properties and stability of the nanocomplexes. Subsequently, the nanocomplexes were characterized using scanning electron microscopy (SEM) and spectroscopic techniques. We further investigated its drug release behavior in simulated gastrointestinal fluids. Additionally, a DSS-induced murine ulcerative colitis (UC) model was utilized to evaluate the therapeutic efficacy of Zein/FA–PEC/CS nanocomplexes against colitis. Parallel assessments of gut microbiota modulation were conducted to determine their potential therapeutic applications. This nano-delivery system effectively enhanced the oral bioavailability of ferulic acid (FA) and will enable more precise controlled release of FA in vivo. These findings establish a theoretical foundation for FA-assisted pharmacological interventions in UC management while proposing innovative strategies for colon-targeted delivery system development.

## 2. Materials and Methods

### 2.1. Materials

FA (purity ≥ 99%), Zein, CS (medium viscosity), and PEC (galacturonic acid [dry base] ≥ 74.0%) were purchased from Shanghai Aladdin Reagent Co., Ltd. (Shanghai, China). Ethanol (purity ≥ 99.5%) was purchased from Shanghai McLean Biochemical Technology Co., Ltd. (Shanghai, China). Hydrochloric acid (purity ≥ 99.5%) was purchased from Xilong Science Co., Ltd. (Shantou, China). Simulated gastric fluid (SGF) and simulated intestinal fluid (SIF) were purchased from Feijing Biotechnology Co., Ltd. (Fuzhou, China).

### 2.2. Preparation of Nanocomplexes

The Zein/FA–PEC/CS complex nanoparticles were prepared using antisolvent precipitation method according to Yuan’s description [25]. Simply, The PEC/CS solution was prepared by dissolving equal amounts of CS (0.5 wt%) and PEC (0.5 wt%) in 0.1 mol/L HCl and stirring for 2–3 h with a magnetic stirrer to allow for complete dissolution. Similarly, Zein/FA solution was obtained by dissolving equal amounts of Zein and FA in 70% (*v*/*v*) ethanol and stirring with a magnetic stirrer for 10–20 min for complete dissolution. The Zein/FA solution was dropped into the PEC/CS solution at a specific ratio, and Zein/FA (core)–CS/PEC (shell) polymers were gradually formed and precipitated from the solution as the ethanol concentration decreased. The precipitates were collected via centrifugation and freeze-dried to obtain the final nanoparticles. Nanocomplexes prepared at volume ratios of 1:9, 3:7, 6:4, and 5:5 (PEC/CS solution: Zein/FA solution) were designated as Y1, Y2, Y3, and Y4, respectively.

### 2.3. Particle Size and ζ-Potential Measurements

The samples were diluted to 0.1 wt% using deionized water, followed by centrifugation and sonication of the supernatant for 1 h. The average particle size, polydispersity index (PDI) and zeta potential were obtained using a Zetasizer (Malvern Instrument, Great Malvern, UK). Each sample was replicated three times, and the mean values were calculated.

### 2.4. EE and LC

The prepared Zein/FA–PEC/CS complex solution was centrifuged (10,000× *g*, 20 min), and the precipitate was washed using ethanol to remove free FA crystals. The amount of free FA in the supernatant was determined at 320 nm using a UV–visible spectrophotometer (TU-1810, Persee, Beijing, China). Particularly, the standard FA was dissolved in ethanol to prepare a standard solution of FA with a purity of 0~12 μg/mL to establish a standard curve. The EE and LC were calculated using Equation (1) and Equation (2), respectively, based on the previously established method [16].

(1) $$\mathrm{EE}\%=\frac{total\;FA-free\;FA}{total\;FA}\times 100$$

(2) $$\mathrm{LC}\%=\frac{total\;FA-free\;FA}{weight\;of\;PEC/CS-Zein\;complexes}\times 100$$

### 2.5. Scanning Electron Microscopy (SEM)

The freeze-dried samples were affixed with double-sided adhesive, mounted on stainless steel, and sprayed with gold before observation. Then, the microscopic surface morphology of these samples was photographed using a scanning electron microscope (Thermo Fisher Scientific, Waltham, MA, USA).

### 2.6. Fourier Transform Infrared Spectroscopy (FTIR)

The structural characteristics of PEC, CS, Zein, FA powders, and freeze-dried nanocomplexes were investigated using FTIR (Thermo Nicolet IS5, USA). The samples were mixed with KBr at a mass ratio of 1:100 (sample:KBr) and pressed into a pellet. The readings were taken with wavelengths of between 400 and 4000 cm^−1^ and analyzed using an OMNIC, software version 8.0.

### 2.7. Circular Dichroism Spectroscopy (CD)

CD spectra were recorded using a CD spectrometer (JASCO, Tokyo, Japan). The scan range was set to 190–260 nm, and the test temperature was 25 °C. The samples were diluted to 0.1 mg/mL with ultrapure water and scanned three times before the assay. Finally, calculations of the secondary structure of the samples were performed using CDNN 2.1 software (Applied Photophysics, Ltd., Leatherhead, UK).

### 2.8. Differential Scanning Calorimetry (DSC)

The thermal denaturation temperature of the samples was determined using a DSC (DSC250, TA Instruments, New Castle, DE, USA). A sample (5 mg) was added to a sealed aluminum crucible, and the heat flow versus temperature profile of the sample was recorded as it was heated from 30 to 200 °C at 10 °C/min.

### 2.9. Storage Particle Size Stability

The Zein/FA–PEC/CS freeze-dried nanoparticles were redissolved, and the solution was stored in a 4 °C refrigerator for 31 days. A volume of 1.5 mL of solution was utilized every 10 days to assess particle size during the storage duration.

### 2.10. In Vitro Release of Zein/FA-PEC/CS Nanocomplexes

In vitro simulated digestion of samples was performed as previously described [26]. Briefly, 0.02 g of Zein/FA–PEC/CS nanocomplexes (Y3) was accurately weighed and placed in a 100 mL conical flask. Then, 20 mL of SGF was added, and the flask was placed in a 37 °C constant temperature oscillator at a frequency of 100 rpm for 2 h. Then, 20 mL of SIF was added to the conical flask and placed in a constant temperature shaker at 37 °C for 3 h at a frequency of 200 rpm. Equal amounts of digest were extracted from the reaction system at an appropriate time interval to assess the cumulative release rate of FA. Equal amounts of the gastrointestinal simulated solution were supplemented at the same time. Gastrointestinal simulant was used as a blank control, and the cumulative release was calculated according to Equation (3):

(3) $$\mathrm{Cumulative}\mathrm{release}\;(\%)=\frac{Ve\times \sum_{1}^{n-1}Ci+Vo\times Cn}{m}\times 100$$

where *Ve* is the volume of each sample, *Vo* is the total volume of simulated gastrointestinal fluid, *Ci* is the FA concentration in the simulated gastrointestinal fluid at the ith replacement sampling, *m* is the mass of FA in nanocomplexes, and *n* is the number of times the simulated gastrointestinal fluid is replaced.

### 2.11. Animal Treatments and Experiments

#### 2.11.1. Mice Treatment and Drug Administration

C57BL/6J mice (Male, 6–8 weeks, 20 g ± 2 g) were bought from Hunan SJA Laboratory Animal Co., Ltd. (Changsha, China). All mice (48) were maintained at a temperature of 22 ± 3 °C and subjected to a 12 h dark–light cycle. They were given unrestricted access to sustenance and distilled water. The research scheme was approved by Hainan University Animal Ethics Committee (approval number HNUAUCC-2021-00109). Based on prior investigations, we established the optimal dosage range for orally administered therapeutics in C57BL/6j mice [6,10,11], wherein the effective dose of encapsulated FA was determined based on the loading capacity. Following one week of acclimation feeding, the mice were randomly allocated into six groups (*n* = 8): Control (standard diet and water), DSS (standard diet and DSS water), SASP (positive control, standard diet and DSS water with 200 mg/kg SASP per day), FANL (low dose, standard diet and DSS water with 100 mg/kg Zein/FA–PEC/CS per day), FANH (high dose, standard diet and DSS water with 200 mg/kg Zein/FA–PEC/CS per day), FA (standard diet and DSS water with 100 mg/kg FA per day). The mice were fed 3% DSS water for 7 days. The body weight of the mice in each group was recorded daily, and fecal samples were collected for analysis of the fecal microbiome on the last day before sacrifice. After the last administration, all of the mice were fasted for 12 h. The mice were anesthetized with isoflurane, and blood was taken from the eyes on day 8, followed by cervical dislocation and execution. The eye blood was placed at room temperature for 1 h and centrifuged (3000× *g*, 15 min) to obtain the serum. The mice were dissected, and the colon length and spleen weight were recorded. Finally, one part of the collected colon was placed in formalin for pathologic sectioning, and the other part was preserved in liquid nitrogen and stored at −80 °C for subsequent use.

#### 2.11.2. Clinical Observations and Organometric Indices

The body weight, diarrhea, blood in stool, activity status, and death of the mice were observed and recorded daily. The disease activity index (DAI) was evaluated according to the DAI scoring rules (Table 1). The splenic index was calculated using Equation (4).

Splenic index = Spleen weight (mg)/Mouse body weight (g)(4)

#### 2.11.3. Histopathological Examination

Colon tissues were fixed with 10% formalin and were sliced to a thickness of 5 mm after embedding using paraffin. Subsequently, the slices were stained and observed after staining with hematoxylin and eosin (H&E) and Periodic Acid-Schiff stain (PAS), respectively.

#### 2.11.4. Determination of Biochemical Indices In Serum and Colonic Tissues

Inflammatory cytokines (IL-1b, TNF-α, and IL-10) in serum were detected using ELISA kits following the manufacturer’s protocol, purchased from Wuhan Huiyucheng Biotechnology Co., Ltd. (Wuhan, China). Colonic tissues were homogenized in saline, then the glutathione (GSH), Nitric Oxide (NO), Superoxide dismutase (SOD), and malondialdehyde (MDA) contents in the colon were determined using kits obtained from Nanjing Jiancheng Bioengineering Institute following the manufacturer’s protocols.

#### 2.11.5. Gut Microbial Analysis

Fecal samples were taken from the −80 °C freezer for gut microbiota abundance analysis. Simply, DNA was extracted using an ALFA-SEQ Magnetic Stool DNA Kit (DC305-08, Guangzhou, China) based on the manufacturer’s instructions. DNA purity and concentration were measured using a Nanodrop One instrument (Thermo Fisher Scientific, MA, USA). The V3-V4 variable regions of the microbial DNA were amplified with barcode-specific primers and Premix Taq. Library construction was performed following the standard protocol of the ALFA-SEQ DNA Library Prep Kit. The library fragment size was evaluated using the Qsep400 high-throughput nucleic acid–protein analysis system, while the library concentration was measured with a Qubit4.0 fluorometer (Thermo Fisher Scientific, Waltham, USA). The constructed amplicon libraries were sequenced on Illumina platforms using PE250 chemistry. The microbial communities were analyzed using the Illumina MiSeq platform according to standard protocols, conducted by Guangdong Magigene Biotechnology Co., Ltd., Shenzhen, China. Finally, the sequencing results were obtained via the Magigene company’s cloud platform (cloud.magigene.com, accessed on 29 November 2024).

### 2.12. Statistical Analysis

All data are presented as means ± standard deviation (SD). The data were analyzed using IBM SPSS 27 software. Normality and homoscedasticity tests were performed, followed by either one-way ANOVA or Welch’s ANOVA. Duncan’s multiple range test was used to determine significant differences among groups. The significance level was *p* < 0.05. The experimental results were analyzed and plotted using Origin 2024 software.

## 3. Results and Discussion

### 3.1. Particle Size and ζ-Potential

**Figure 1:**
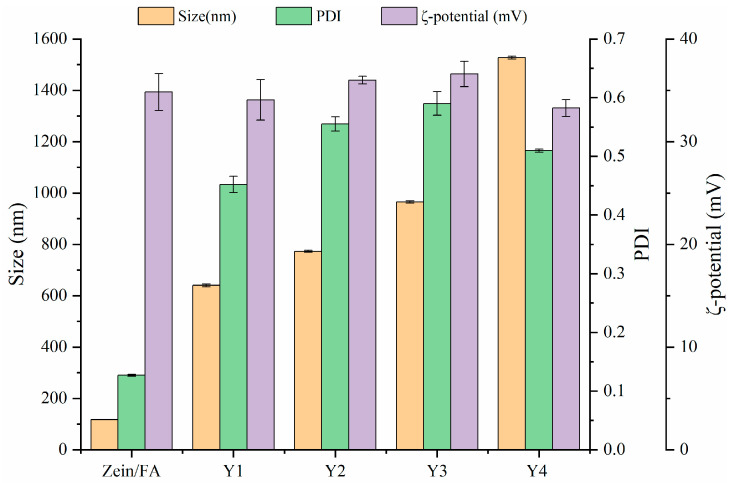
The particle size, PDI, and ζ-potential of Zein/FA and Zein/FA–PEC/CS nanocomplexes for different PEC/CS–Zein/FA volume ratios.

The particle size, ζ-potential, and PDI of Zein/FA and Zein/FA–PEC/CS nanoparticles are shown in Figure 1. The results revealed that Zein/FA had the smallest particle size of 117.6 nm. When PEC/CS was added to the system, the particle size of the nanoparticles increased dramatically. This was due to the strong hydrophilicity of PEC/CS, which helps Zein/FA to become embedded by PEC/CS, forming a core–shell structure. As expected, the ratio of PEC/CS input in the system increased and the nanoparticle size kept increasing. Zeta potential > ±30 mV is usually used as an approximate threshold for solution stability [27]. The zeta potentials of the five nanoparticles were all greater than +30 mV, indicating that the system had good stability and was not easy to aggregate. Among them, Y3 had the highest zeta potential of 36.6 mV, indicating that the Y3 nanoparticles were the most stable. Zein/FA had the smallest PDI of 0.127 and good dispersion with uniform solution size. Following the introduction of PEC/CS into the system, the PDI of the solution increased to 0.5–0.6. This might have been due to the formation of core–shell structures with uneven shells, which leads to an increase in the size of nanoparticles and an increase in the density of the solution. Consequently, the system exhibited heightened viscosity and compromised dispersion stability.

### 3.2. EE and LC

**Figure 2:**
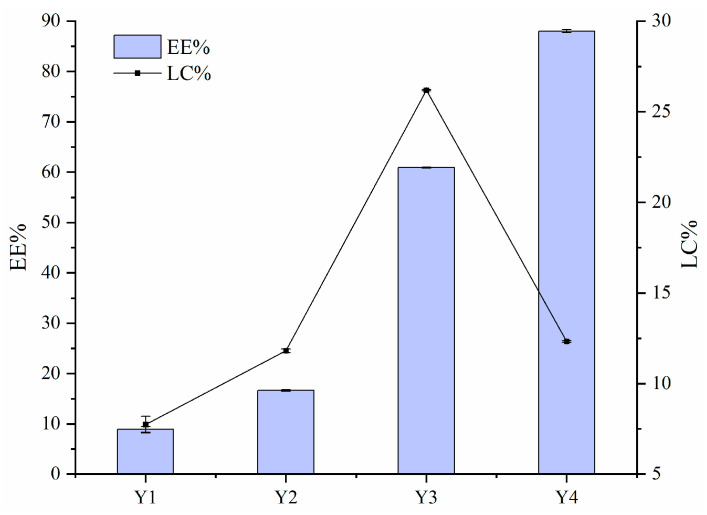
The encapsulation efficiency (EE) and loading capacity (LC) for FA of Zein–PEC/CS complexes.

Figure 2 illustrates the EE and LC values of FA encapsulated by Zein–PEC/CS complexes. The data show that the EE values decreased with increasing FA concentration. This reduction was likely due to excessive Zein/FA content in the system, which exceeded the loading capacity of PEC/CS. The binding sites between Zein/FA and PEC/CS reached saturation at this point due to the aggregation of nanoparticles, which was thought to be related to the relatively minimal zeta potential in this phase of the system. Given that the equal total mass of Zein–PEC/CS was introduced into the Y1–Y4 systems, the Y3 nanoparticles demonstrated the highest FA loading capacity.

### 3.3. Micromorphology of Complex Nanoparticles

**Figure 3:**
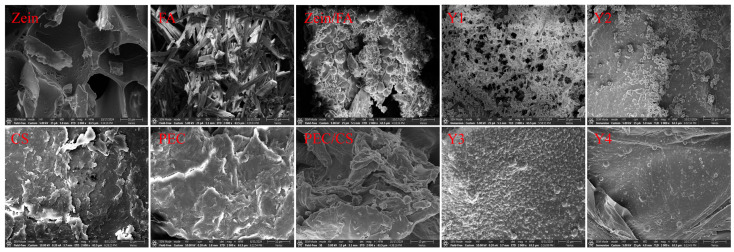
SEM images of all samples. All SEM images have a scale bar of 10 μm and were magnified at 2000×.

As shown in Figure 3, Zein exhibited a porous lamellar structure with irregularly distributed voids [28]. Furthermore, this porous lamellar architecture provided a high specific surface area, facilitating enhanced dispersion and dissolution, which makes it highly suitable for the manufacturing of nanoparticles and the preparation of thin films. FA crystals displayed needle-like, rod-shaped morphologies with smooth surfaces [29]. CS aggregates exhibited sheet-like or fibrous morphologies with irregular edges. The high specific surface area resulting from the flaky stacking and microporous structure of the CS surface enhances the suitability for drug loading and adsorption applications. PEC exhibited an irregular sheet-like morphology with rough surfaces. Visible cracks and honeycomb-like pores were observed, which endowed superior water-retention properties. SEM analysis revealed that the porous Zein matrix effectively embedded FA particles, resulting in Zein/FA exhibiting polydisperse spherical morphologies [30]. In contrast, the PEC/CS composite exhibited an irregular configuration.

The SEM images consistently revealed well-defined spherical morphologies for all Zein/FA–PEC/CS nanoparticles. Y1 exhibited the smallest particle size but formed aggregates with pronounced interparticle adhesion, which was thought to be due to excess FA interacting with the polysaccharide through hydrogen bonding, which disrupts the nanoparticles’ inherent surface smoothness. Meanwhile, a fraction of unbound FA molecules adhered to the nanoparticle surfaces, exacerbating the aggregation tendency. Y2 nanoparticles displayed smooth surfaces and good dispersibility. Y4 nanoparticles were relatively dispersed but showed non-uniform particle sizes. In contrast, Y3 nanoparticles had the most uniform size, the most regular spherical shape, and the most uniform distribution. The large ζ-potential made the distribution of particles more extensive [16]. The SEM results confirmed the formation of a uniform micromorphology and the effective encapsulation of FA by Zein–PEC/CS complexes.

### 3.4. FTIR Analysis

**Figure 4:**
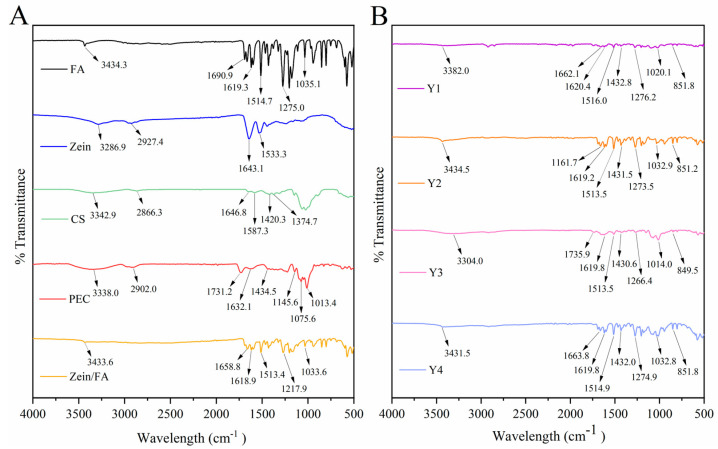
(**A**) FTIR spectra of FA, Zein, CA, PEC, and Zein/FA nanocomplexes, (**B**) FTIR spectra of Zein/FA−PEC/CS nanocomplexes for different PEC/CS−Zein/FA volume ratios.

Figure 4 displays the FTIR spectra of all the samples. The -OH peak of Zein/FA shifted to 3433.64 cm^−1^, indicating that hydrogen bonding interaction occurs in the nanocomplexes. Two characteristic protein peaks of Zein were amide I (C=O stretching vibration and N-H deformation vibration) at 1643.1 cm^−1^ and amide II (C-N stretching vibration and N-H bending vibration) at 1533.3 cm^−1^ [31]. The blue shift of the amide I band of Zein/FA compared with Zein indicated alterations in the secondary structure of the protein [32]. The content of α-helix decreased and the content of β-sheet increased [8]. The peak at 1690.9 cm^−1^ in FA was attributed to the C=O (-COOH) stretching vibration, while peaks at 1619.3 cm^−1^ and 1514.7 cm^−1^ were attributed to C=C stretching vibrations in benzene rings [33]. Peaks at 1275.0 cm^−1^ and 1035.1 cm^−1^ were attributed to C-O-C vibrations. Peaks at 1618.93 cm^−1^ and 1513.41 cm^−1^ in the Zein/FA nanocomplexes were attributed to C=C stretching vibrations of FA. Peaks at 1271.92 cm^−1^ and 1033.64 cm^−1^ were attributed to C-O-C vibrations in FA. These peaks shifted slightly along with a significant reduction in intensity. This indicated that FA was encapsulated within the hydrophobic domains of Zein or engaged in hydrophobic interactions, which shielded the vibrations of these chemical bonds [34].

The shift of the -OH peak of Y1–Y4 in the 3300–3500 cm^−1^ region indicated the formation of hydrogen bonds [8,35,36,37]. Amide I exhibited a further blue shift or disappearance compared with Zein, suggesting alterations in the protein’s secondary structure, potentially involving a transition from α-helix to β-sheet conformations [8]. Peaks near 1432 cm^−1^ in Y1–Y4 likely arose from C-O (-COOH) in PEC [16]. Peaks near 1619 cm^−1^ and 1514 cm^−1^ arose from C=C in the benzene rings of FA. Peaks near 1275 cm^−1^ and 1035 cm^−1^ arose from C-O-C in FA [33,38]. Additionally, new peaks emerged around 850 cm^−1^ in these samples, which may correlate with para-substitution in the benzene rings of FA. Collectively, the nanocomplexes formed through varying ratios of Zein, FA, CS, and PEC resulted in weakened characteristic peaks of individual components, particularly in hydroxyl, amide, and carboxylic acid regions. This attenuation likely stemmed from interactions mediated by hydrogen bonding, electrostatic forces, and hydrophobic effects [32,35].

### 3.5. CD Analysis

**Figure 5:**
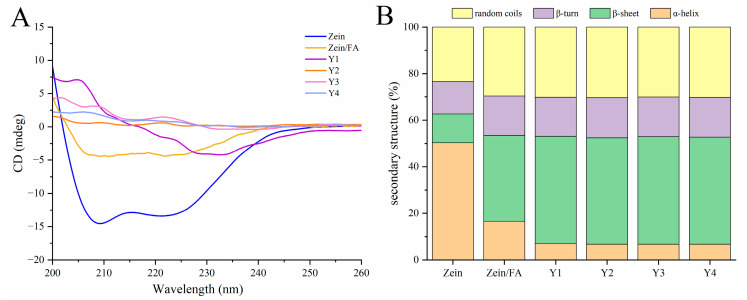
(**A**) CD spectroscopy of Zein, Zein/FA, and Zein/FA–PEC/CS nanocomplexes for different PEC/CS–Zein/FA volume ratios. (**B**) The secondary structure contents.

As shown in Figure 5A, the addition of FA and PEC/CS altered the secondary structure of Zein. This structural modification affected ellipticity peaks at 195 nm (β-sheet) and 208 nm (α-helix). As shown in Figure 5B, the secondary structure percentage orders of the native Zein were as follows: α-helix > random coil > β-turns >β-sheets, demonstrating that the predominant structure of Zein is α-helices. The content of α-helices and β-sheets of natural Zein was 50.30% and 12.37%, respectively. Upon binding with FA, the α-helix content significantly decreased to 16.52%, while β-sheets increased to 36.87%. Subsequent complexation of Zein/FA with PEC/CS further reduced the α-helix content and increased the β-sheet content, accompanied by a rise in random coil structures. The above results demonstrated that the formation of Zein/FA complexes altered protein secondary structure, inducing the transition of α-helices and β-sheets, which aligned with the findings reported by Wang et al. [8]. The α-helix was primarily stabilized by intermolecular hydrogen bonds formed between C=O and N-H. The incorporation of FA would disrupt hydrogen bond formation, hence influencing the α-helix composition of Zein [39]. The addition of PEC-CS further reduced the ordered α-helical structures of the protein, indicating hydrogen bond breakage or reorganization. This may enhance the interface compatibility with PEC/CS, expose the hydrophobic core of protein, and promote helical unfolding to facilitate nanocomplex assembly. The Y1–Y4 samples exhibited minimal differences in protein secondary structure, likely attributable to the formation of thermodynamically stable assembly patterns between PEC/CS and Zein/FA across varying ratios. The protein–polysaccharide interactions may have approached saturation under these conditions.

### 3.6. DSC Analysis

**Figure 6:**
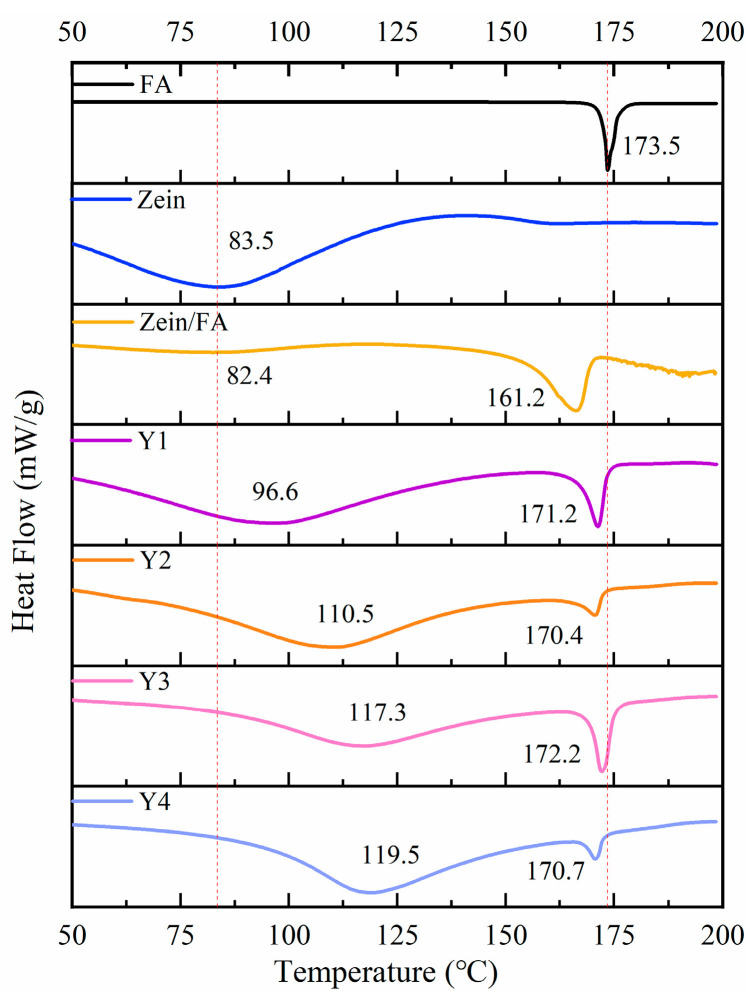
DSC thermograms of FA, Zein, Zein/FA, and Zein/FA–PEC/CS nanocomplexes for different PEC/CS–Zein/FA volume ratios.

As shown in Figure 6, FA displayed a sharp endothermic peak at 173.5 °C, indicating its melting temperature (Tm) at 173.5 °C. Zein displayed an endothermic peak at 83.5 °C, indicating its denaturation temperature (Td) at 83.5 °C. Upon Zein and FA forming nanoparticles, Zein exhibited a marginal decrease in Td to 82.4 °C and a pronounced decrease in Tm to 161.2 °C. The decrease in Td likely arose from hydrophobic interactions between Zein and FA, which induced structural destabilization of the protein [8,40]. The reduction in Tm may have resulted from the encapsulation of FA within Zein’s hydrophobic environment, which disrupted the crystalline structure of native FA and led to a less ordered molecular arrangement than pure FA. Additionally, Zein likely interfered with hydrogen bonding interactions among FA molecules, further reducing thermal stability. In summary, the addition of PEC/CS significantly increased the Td of Zein, which enhanced the thermal stability of the nanocomplexes.

### 3.7. Storage Particle Size Stability

**Figure 7:**
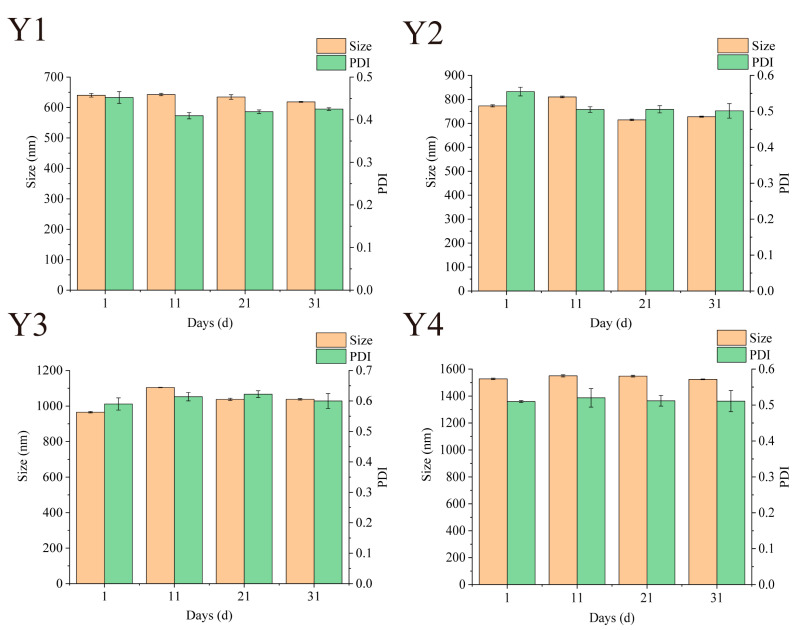
Effects of different storage times on particle size and PDI of Zein/FA–PEC/CS nanocomplexes for different PEC/CS–Zein/FA volume ratios.

Maintaining stability during storage is a prerequisite for nano-systems to achieve commercial applications [16]. The stability of Zein/FA–PEC/CS nanocomplexes can be assessed by examining the variations of particle size and PDI. As shown in Figure 7, the particle size and PDI of Zein/FA–PEC/CS nanocomplexes fluctuated slightly during 31 days of storage at 4 °C. The PDI values remained within the range of 0.08–0.7, indicating that the nanocomplex system maintained a relatively uniform particle size distribution during storage. After 31 days of storage, only Y3 sample size increased by 75.6 nm. During storage, no samples exhibited turbidity or discernible flocculation, indicating that the Zein/FA–PEC/CS nanocomplexes possess excellent storage stability for practical applications.

### 3.8. Release Kinetics

**Figure 8:**
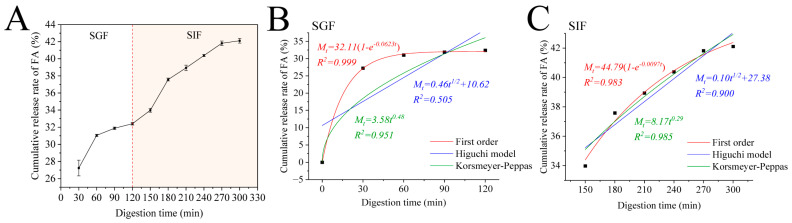
(**A**) The cumulative release of FA from Zein/FA−PEC/CS complex nanoparticles in simulated gastrointestinal tract. (**B**) Kinetic model fitting results of Zein/FAPEC/CS nanocomplexes in SGF. (**C**) Kinetic model fitting results of Zein/FA−PEC/CS nanocomplexes in SIF.

Encapsulating FA within Zein–PEC/CS nanocomposites may mitigate its gastric losses, ensuring its delivery to the intestinal tract for optimal digestion and absorption. Therefore, we investigated the cumulative release profiles of Zein/FA–PEC/CS nanocomplexes in SGF and SIF. As shown in Figure 8A, the Zein/FA–PEC/CS nanocomplexes exhibited low FA release in SGF. After 2 h of simulated gastric digestion, the cumulative release of FA reached only 32.41%. However, the cumulative release increased significantly within the first 60 min and plateaued when transferred to SIF. It ultimately achieved 42.1% cumulative release by 300 min. This demonstrated that the coacervate layer formed by the Zein–CS/PEC complexes exhibited stability, which effectively protected FA from gastric acid degradation while delaying FA release from the Zein/FA–PEC/CS nanocomplexes in the intestinal tract.

To further investigate the release mechanism of protein–polysaccharide nanocomplexes in the simulated gastrointestinal tract, this study employed the first-order kinetic model, Higuchi model, and Korsmeyer–Peppas model to fit the release profiles of the nanocomplexes. The fitting results are displayed in Figure 8B,C. The release kinetic parameters are shown in Table 2. In the SGF phase, the first-order kinetic model demonstrated the best fitting performance, with *R*^2^ values of 0.999. In the SIF phase, the Korsmeyer–Peppas model demonstrated the best fitting performance, with *R*^2^ values of 0.985. The data obtained revealed that the release behavior of FA in the Zein/FA–PEC/CS nanocomplex system was governed by first-order kinetics in the SGF phase and Fickian diffusion in the SIF phase. The Zein/FA–PEC/CS nanocomplex achieved sustained release through a diffusion-controlled mechanism, making it suitable for designing sustained-release or targeted delivery systems.

### 3.9. Zein/FA–PEC/CS Alleviated the Symptoms of UC Induced by DSS in Mice

**Figure 9:**
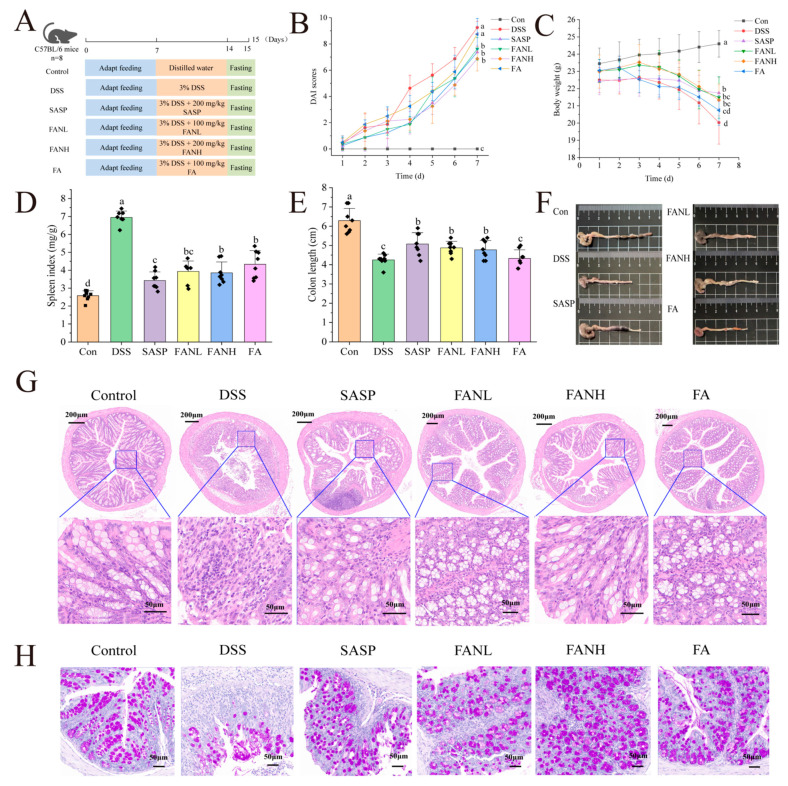
Effects of Zein/FA–PEC/CS on DSS-induced colitis mice. (**A**) Schematic diagram of the experimental design. (**B**) Body weight. (**C**) DAI scores. (**D**) Spleen index. (**E**) Colon length. (**F**) Representative images of the colon. (**G**) H&E staining. (**H**) PAS staining. The data are expressed as means ± SD (*n* = 8). Data with different letters are significantly different (*p* < 0.05).

The intervention effect of oral Zein/FA–PEC/CS nanocomplexes on colitis was evaluated using a DSS-induced UC mice model. Figure 9A shows the experimental process. The most common clinical indicators of UC were weight loss, shortened colon length, and bloody feces [41]. The DAI was evaluated by assessing the scores of fecal consistency, occult blood in feces, and weight loss in mice, and the final composite score was used to determine the severity of the disease in mice. After free access to DSS, the DSS group’s weight began to drop significantly on day 3, and DAI scores started to increase rapidly (Figure 9B,C). On day 7, the DSS group’s weight was significantly lower than the Con group, and DAI scores were significantly higher than the Con group. The symptoms of the mice in the other treatment groups were all improved. The body weight of the FA group mice was only slightly higher than the DSS group, and their DAI scores were only slightly lower than the DSS group, which was considered to be due to the degradation of orally administered unencapsulated FA during its passage through the digestive tract of the mice. This degradation occurred due to the pH levels and enzymes present in the digestive juices at various stages. Research has shown that DSS-induced UC mice exhibit splenomegaly [6], and a similar phenomenon also occurred in this study (Figure 9D). The FANL and FANH groups significantly alleviated the symptoms of weight loss in mice, improved their DAI scores, and reduced their spleen indices. Furthermore, the FANL and FANH groups effectively increased the colon length (Figure 9E,F). In conclusion, these findings demonstrate that the Zein/FA–PEC/CS nanocomplexes exhibited a significant alleviating effect on DSS-induced colitis in mice.

### 3.10. Zein/FA–PEC/CS Improved the Histological Changes of the Colon Tissue

To further confirm the alleviating effects of Zein/FA–PEC/CS nanocomplexes on UC, histopathological analysis of the mice colon was conducted using H&E and PAS staining (Figure 9G,H). The Con group mice exhibited normal colon tissue structure, characterized by dense columnar epithelium, intact intestinal glands, and clear stratification between the mucosa and submucosa. In contrast, the DSS group mice displayed severe colon damage, including incomplete epithelium, erosion, and lesions in the crypts. The tissue margins were unclear, indicating severe tissue damage, loss of goblet cells, reduced mucus, and infiltration of inflammatory cells into the muscle layer. Additionally, the missing glands confirmed the successful establishment of the UC model. In all intervention groups, the UC mice exhibited alleviated inflammation in the colonic mucosa, with restored epithelial cell structures. The FANH group showed particularly significant effects, with intestinal tissue sections showing normalization of the intestinal structures, intact crypt structures, and numerous cup cells. These intestinal features were similar to those observed in the Con group mice, which indicated that the high-dose Zein/FA–PEC/CS nanocomplexes exhibited a significant alleviating effect on DSS-induced intestinal damage in mice.

### 3.11. Zein/FA–PEC/CS Modulates Inflammatory Cytokine Secretion and Oxidative Stress in Colitis Mice

**Figure 10:**
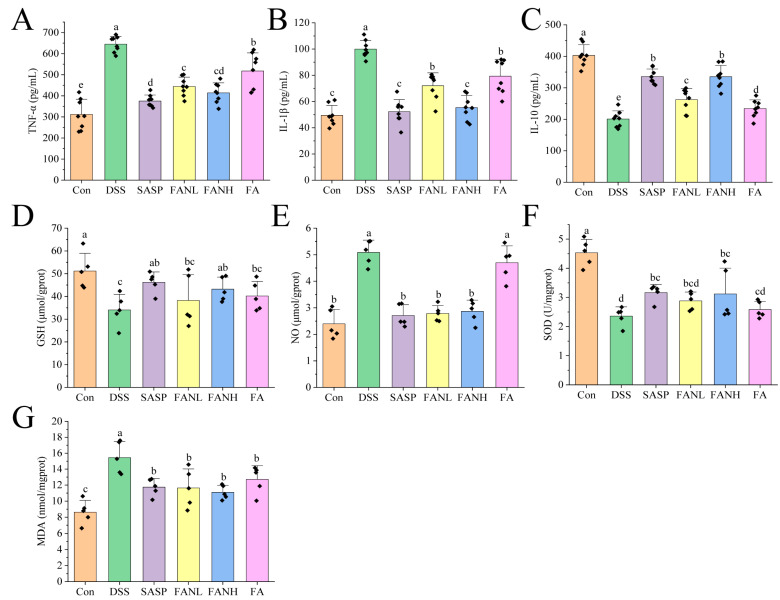
Effects of Zein/FA–PEC/CS nanocomplexes on inflammation and oxidative stress. Effects of Zein/FA–PEC/CS nanocomplexes on (**A**) interleukin-1β (IL-1β), (**B**) tumor necrosis factor alpha (TNF-α), and (**C**) interleukin-10 (IL-10) in mice serum. Effects of Zein/FA–PEC/CS nanocomplexes on (**D**) glutathione (GSH), (**E**) nitric oxide (NO), (**F**) malondialdehyde (MDA), and (**G**) superoxide dismutase (SOD) in the colons of mice. Data are expressed as means ± SD (n =5 or 8). Data with different letters are significantly different (*p* < 0.05).

The severity of UC is directly related to the levels of inflammation-related cytokines. Therefore, measuring cytokines such as IL-1β, TNF-α, and IL-10 can reflect the state and severity of inflammation [42]. TNF-α and IL-1β are pro-inflammatory factors expressed by M1 macrophages, while IL-10 is an anti-inflammatory factor secreted by M2 macrophages [43]. Compared to the Con group, the levels of pro-inflammatory factors IL-1β and TNF-α in the colon tissue in the DSS group were significantly increased (Figure 10A,B). Meanwhile, the level of the anti-inflammatory factor IL-10 was significantly decreased in the DSS group (Figure 10C), which indicated that the DSS induced severe inflammation in the mice. In the other intervention groups, the levels of pro-inflammatory factors were significantly lower compared to the DSS group, while the levels of anti-inflammatory factors were significantly higher. Studies have shown that FA can reduce the levels of pro-inflammatory factors, increase the levels of anti-inflammatory factors, and improve colitis [10,11,14,44]. Notably, the FANH group significantly reduced the levels of pro-inflammatory factors in the serum of mice and significantly increased the levels of anti-inflammatory factors compared to the FA group. The above data imply that the Zein/FA–PEC/CS nanocomplexes better preserved the biological activity of FA and further enhanced its anti-inflammatory capability.

In UC, inflammation and oxidative stress are closely related, and their synergistic effects promote the progression of UC. GSH, NO, SOD, and MDA are commonly used as universal markers to assess oxidative damage [45,46]. GSH is a crucial intracellular antioxidant that protects intestinal mucosa by scavenging reactive oxygen species (ROS) and maintaining redox homeostasis [47]. In UC patients, decreased GSH levels result in impaired antioxidant defense capacity and exacerbated oxidative damage. NO regulates vasodilation and mucosal blood flow to maintain intestinal barrier function. However, overproduction results in protein nitration, DNA damage, and apoptosis. In UC patients, NO levels are significantly elevated in both colonic tissues and serum [14]. SOD is a bioactive substance capable of eliminating harmful substances produced during organism metabolism. Reduced SOD activity in the colonic mucosa of UC patients lead to ROS accumulation [3,48]. MDA is a typical product of lipid peroxidation and may be associated with increased mucosal damage and intestinal inflammation [47]. Compared to the Con group, the DSS group showed significantly reduced levels of GSH and SOD (Figure 10D,E) and significantly increased levels of NO and MDA (Figure 10F,G), which indicated that the mice exhibited a pronounced oxidative stress response. All intervention groups alleviated oxidative stress in the mice. Compared to the DSS group, the FANL group exhibited 12.3% and 22.1% increases in GSH and SOD levels, respectively, while the FANH group showed more pronounced elevations of 26.8% in GSH and 32.2% in SOD. Conversely, NO and MDA levels decreased by 45.2% and 24.7% in the FANL group relative to DSS controls, with the FANH group demonstrating 43.6% NO reduction and 28.2% MDA decline. In comparison, the FANL group and FANH group exhibited superior therapeutic effects compared to the unencapsulated FA group. The FANH group demonstrated better therapeutic effects than the FANL group. These results indicate that the Zein/FA–PEC/CS nanocomplex demonstrates a superior ability to alleviate oxidative stress compared to unencapsulated FA.

### 3.12. Modulation of the Intestinal Bacteria by Zein/FA–PEC/CS

**Figure 11:**
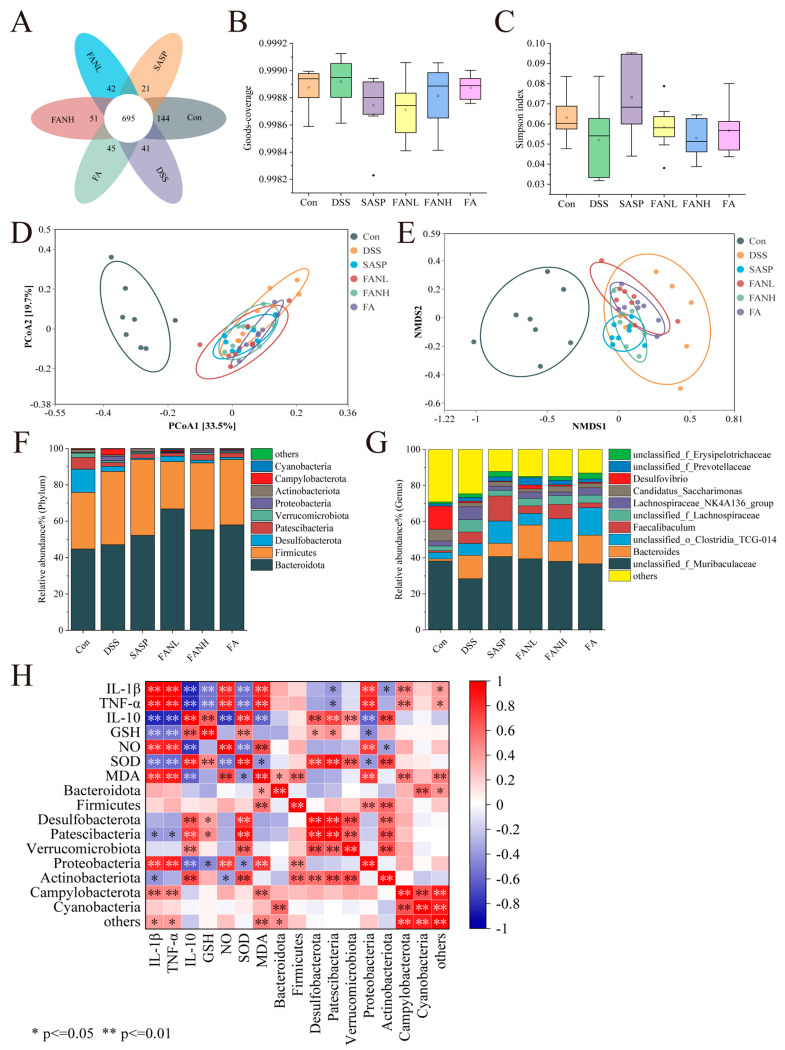
Effects of Zein/FA−PEC/CS nanocomplexes on gut microbiome structure of colitis mice. (**A**) Venn diagram of OTUs. (**B**) Goods−coverage index. (**C**) Simpson index. (**D**) PCoA analysis at the genus level. (**E**) NMDS analysis at the genus level. (**F**) The relative abundance of species at the phylum level. (**G**) The relative abundance of species at the family level. (**H**) Correlation analysis for UC−related indicators and the gut microbiota at the phylum level.

Operational Taxonomic Units (OTUs) are artificially defined classification units used for cluster analysis of biological samples using molecular biological methods, where sequences with greater than 97% similarity are clustered as OTUs. The Venn diagram visually demonstrated the similarity and overlap of OTU composition among sample groups (Figure 11A). The OTU numbers for the Con group, DSS group, SASP group, FANL group, FANH group, and FA group were 1042, 971, 903, 968, 994, and 985, respectively, with 695 shared OTUs. Goods-coverage and the Simpson index revealed alpha diversity. Goods-coverage indicated that the sequencing depth of each group was sufficient (Figure 11B). The DSS group showed a decrease in the Simpson index, suggesting reduced gut microbial diversity (Figure 11C), while other intervention groups exhibited varying degrees of recovery in gut microbial diversity. Principal coordinate analysis (PCoA) and non-metric multidimensional scaling (NMDS) were used to assess beta-diversity. The result of PCoA revealed significant separation between the control and the DSS groups, indicating marked differences in the microbial composition between the DSS and the control groups. Based on beta-diversity analysis, the SASP group, FANL group, FANH group, and FA group improved the intestinal microbiota of the mice. Furthermore, consistency was observed between the results of PCoA and NMDS.

At the phylum level, *Bacteroidota* and *Firmicutes* were the two most relatively abundant bacterial phyla, accounting for over 75% of the total (Figure 11F). The intake of DSS led to an increased abundance of *Campylobacterota* and *Proteobacteria* while reducing the abundance of *Desulfobacterota*, *Patescibacteria*, and *Actinobacteria*, consistent with previous results [3,7,41,47,49,50]. *Campylobacterota* and *Proteobacteria* are established detrimental bacteria in UC, capable of disrupting the intestinal epithelial barrier, driving inflammatory responses, and inducing microbial dysbiosis [51]. This has been further confirmed through subsequent correlation analyses between UC-related indices and gut microbiota. They exhibit significantly positive correlations with pro-inflammatory factors and significantly negative correlations with anti-inflammatory factors. The roles of *Desulfobacterota* and *Patescibacteria* in UC require further clarification, whereas *Actinobacteria* predominantly exhibit beneficial effects [52]. The intervention groups exhibited varied restoration of these microbial structural changes. In contrast to previous studies, *Firmicutes* were enriched in the DSS group and other intervention groups [3,7]. This was not only a result of microbial imbalance but might also reflect the selective effect of the host’s inflammatory state on the microbiota. At the genus level, DSS intake increased the abundance of *Bacteroides* and reduced the abundance of *unclassified_f_Muribaculaceae* and *Desulfovibrio* (Figure 11G). *Bacteroides* in UC demonstrate both potential benefits, and under specific conditions, pro-inflammatory detrimental effects, primarily contingent upon the metabolic environment and host immune status. All intervention groups elevated the abundance of *unclassified_f_Muribaculaceae*. The SASP group and FANH group reduced the DSS-induced increase in *Bacteroides* abundance, while the FANL group partially reversed the DSS-induced reduction in *Desulfovibrio* abundance.

Additionally, Spearman’s correlation analysis was performed to further investigate the relationships among inflammatory cytokines, oxidative stress, and the top 10 intestinal microbes at the phylum level in relative abundance (Figure 11H). Most of these indicators showed significant correlations, suggesting they were interconnected and mutually influential. *Campylobacterota* and *Proteobacteria* exhibited significant positive correlations with pro-inflammatory cytokines, NO, and MDA, while showing significant negative correlations with anti-inflammatory cytokines. *Proteobacteria* demonstrated significant negative correlations with GSH and SOD. *Desulfobacterota* displayed significant positive correlations with IL-10 and SOD. *Patescibacteria* showed significant positive correlations with IL-10, GSH, and SOD. *Actinobacteriota* exhibited significant negative correlations with IL-1β and TNF-α, along with significant positive correlations with IL-10 and SOD.

## 4. Conclusions

In this study, Zein/FA–PEC/CS nanocomplexes were successfully prepared. When the volume ratio of PEC/CS to Zein/FA was optimized to 6:4, the nanocomplexes demonstrated favorable encapsulation efficiency (60.1%) and drug loading capacity (26.2%) for FA. Microstructure and spectroscopy analysis confirmed the formation of the nanocomplexes, the driving forces of which mainly involve hydrogen bonding, electrostatic interactions, and hydrophobic interactions. Additionally, the Zein/FA–PEC/CS nanocomplexes demonstrated favorable thermal stability and storage particle size stability, and they achieved sustained release of FA during in vitro digestion. The results of animal experiments confirmed that the nanocomplexes effectively alleviated the symptoms of DSS-induced colitis and attenuated the dysregulation of pro/anti-inflammatory cytokines and antioxidant indicators. Additionally, they partially reversed gut microbial dysbiosis in colitis mice. Moreover, the results demonstrated that the Zein/FA–PEC/CS nanocomplexes exhibited superior efficacy in alleviating colitis symptoms compared to unencapsulated FA, with higher-dose administration demonstrating enhanced therapeutic benefits relative to the lower-dose counterpart. The above findings indicated that the Zein/FA–PEC/CS nanocomplexes exhibited certain potential in addressing therapeutic and nutritional challenges related to colitis.

## Figures and Tables

**Table 1:** Mouse DAI scoring criteria.

Weight Loss (%)	Fecal Character	Degree of Blood in Feces	Score
0	Normal	Negative	0
1–5	Formed, easily adhered	Weakly positive, light yellowish green	1
5–10	Semi-formed, thin and soft	Positive, blue-green	2
10–20	Thin slurry, not adherent to anus	Strongly positive, dark blue-green	3
>20	Diarrhea, adherent to anus	Visible positive, sticky pus, and blood feces	4

**Table 2:** Fitting results of the release kinetic model.

Kinetic Model	Release Mechanism	Equation	*k*		*R* ^2^		*n*	
			SGF	SIF	SGF	SIF	SGF	SIF
First order	First-order release	M_t_/M∞= 1 − e^−k·t^	0.0623	0.0097	0.999	0.983	/	/
Higuchi model	Fickian diffusion	M_t_/M∞ = kt^1/2^	0.46	0.10	0.505	0.900	/	/
Korsmeyer–Peppas	Fickian diffusion (*n* ≤ 0.45); Anomalous transport (0.45 < *n* < 0.89); Case-II transport (*n* = 0.89); Super Case-II transport (*n* > 0.89)	M_t_/M∞ = kt^n^	3.58	8.17	0.951	0.985	0.48	0.29

## Data Availability

The original contributions presented in this study are included in the article. Further inquiries can be directed to the corresponding authors.
